# Unraveling the Enigma: A Five-Year Comprehensive Analysis of Hurthle Cell Tumors in South India's Tertiary Care Center

**DOI:** 10.7759/cureus.57166

**Published:** 2024-03-29

**Authors:** Sumin Sulaiman, Ravindran Chirukandath, Sharath K Krishnan, Niranjana Rajesh, Manoj Antony, Keerthana Mohan, Sowndarya S

**Affiliations:** 1 General Surgery, Government Medical College Thrissur, Thrissur, IND; 2 Surgical Oncology, Government Medical College Thrissur, Thrissur, IND; 3 Surgery, Government Medical College Thrissur, Thrissur, IND

**Keywords:** hurthle cell carcinoma, tumor surgery, adjuvant radiation therapy, thyroid, thyroid nodule size, hurthle cell neoplasm

## Abstract

Background: Hurthle cell tumors of the thyroid gland constitute a rare and enigmatic group of neoplasms, characterized by the presence of Hurthle cells exhibiting abundant eosinophilic cytoplasm and numerous mitochondria. Despite their low incidence, they pose diagnostic challenges and display diverse clinical outcomes. This study aims to provide a comprehensive analysis of the clinicopathological profile of Hurthle cell tumors within a tertiary care center in South India.

Methods: Through a retrospective approach, we analyzed cases of Hurthle cell tumors diagnosed and treated at a tertiary care center over a five-year period. Clinical, radiological, and histopathological data were meticulously collected and scrutinized. The study focused on examining demographic details, presenting symptoms, imaging features, cytological findings, surgical management, and postoperative outcomes of the patients.

Results: A total of 32 cases of Hurthle cell tumors were identified during the study period. The majority of patients were female (84%), with a mean age of 49.6 years for Hurthle cell carcinoma. Thyroid enlargement and neck mass were the most common presenting complaints. Fine-needle aspiration cytology showed characteristic features suggestive of Hurthle cell tumors in 33% of cases. Total thyroidectomy remains the mainstay surgical approach. Histopathological evaluation confirmed 62.5% of cases as benign adenomas and 37.5% as malignant carcinomas. Among malignant cases, 67% showed capsular invasion and 33% demonstrated vascular invasion. Of the patients, 33.3% received adjuvant radiotherapy. The overall survival rate was 100%. In our study, we found that thyroid nodules larger than 3 cm demonstrated a higher propensity for Hurthle cell carcinoma.

Conclusion: Our findings support the multidisciplinary approach in managing Hurthle cell tumors, with a focus on tailored treatment plans for each patient based on individual characteristics. By recognizing the female predominance, assessing nodule size, and employing a combination of thyroidectomy and ablative therapy, clinicians can optimize patient care and contribute to better long-term prognosis and quality of life for those affected by Hurthle cell tumors. Continued research and collaborative efforts are necessary to advance our understanding and refine treatment strategies, paving the way for improved outcomes and enhanced patient management in the future.

## Introduction

Hurthle cell tumors of the thyroid gland, also known as oncocytic tumors, represent a distinct subgroup of thyroid neoplasms. They are characterized by the presence of Hurthle cells, which are large, granular, and eosinophilic cells with abundant mitochondria [1]. Hurthle cell tumors account for approximately 3-5% of all thyroid neoplasms [2], and they can exhibit a spectrum of clinical behaviors ranging from benign adenomas to malignant carcinomas.

Despite their relative rarity, Hurthle cell tumors present diagnostic challenges and therapeutic dilemmas due to their morphological heterogeneity and uncertain biological behavior. The differentiation between Hurthle cell adenoma and carcinoma has been a subject of variation, with researchers employing diverse criteria for this purpose. Additionally, many have proposed different morphologic characteristics to distinguish between benign and malignant Hurthle cell tumors [3]. Recent molecular studies have shed light on the distinct oncogenic features of Hurthle cell carcinomas, suggesting that they represent a separate pathological entity from both Hurthle cell adenomas and follicular cell carcinomas [4,5].

The accurate preoperative diagnosis of these tumors is essential for appropriate management decisions. Fine-needle aspiration cytology (FNAC) is the primary diagnostic modality; however, due to the overlapping cytological features with other thyroid lesions, definitive diagnosis can be challenging [6]. Numerous studies have provided evidence of the effectiveness of fine-needle aspiration (FNA) in identifying Hurthle cell neoplasms among thyroid nodules [7]. However, accurately classifying Hurthle cell neoplasms as either benign or malignant based solely on FNA data can pose challenges [8].

Furthermore, the management of Hurthle cell tumors remains controversial. The optimal extent of surgical resection, the role of adjuvant therapy, and the long-term outcomes in terms of recurrence and survival are subjects of ongoing debate [9]. Studies exploring the clinicopathological features and outcomes of Hurthle cell tumors are crucial for a better understanding of this entity and to guide evidence-based management strategies.

The clinical course of Hurthle cell carcinoma has been a subject of varying interpretations among researchers. Some have reported a very favorable clinical outcome for this tumor, while others have regarded it as a relatively aggressive malignancy of the thyroid gland [10]. This diversity of perspectives highlights the complexity of understanding and managing Hurthle cell carcinoma, warranting further investigation and consensus-building to improve patient outcomes.

This study aims to provide a comprehensive analysis of the clinicopathological profile of Hurthle cell tumors of the thyroid gland in a tertiary care center in South India. By evaluating the demographic characteristics, presenting symptoms, radiological features, cytological findings, histopathological characteristics, surgical management approaches, and postoperative outcomes, we aim to contribute to the existing knowledge regarding the management of Hurthle cell tumors and provide insights into their clinical behavior in our population.

## Materials and methods

The study encompassed a retrospective analysis set in the Department of General Surgery at Government Medical College, Thrissur. Ethical clearance was sought and obtained from the Institutional Ethics Committee, Government Medical College, Thrissur (IEC/GMCTSR/2023/058). It involved meticulous examination of the operative and histopathology records for all Hurthle cell tumor cases operated on an elective basis from January 2018 to March 2023. The aim was to delineate the various subtypes of Hurthle cell tumors and their demographic distribution, including age patterns and sex ratio, clinical presentation, and anatomical locations.

Inclusion and exclusion criteria

The study defined clear inclusion and exclusion criteria to maintain focused research parameters. Included were elective cases diagnosed with Hurthle cell tumors across a wide age range, specifically from 13 to 70 years, that presented to the Department of General Surgery at the said medical college within the designated study period. In our hospital, we only treat patients aged 13 years and above, in general surgery, thus excluding data for younger patients. Conversely, exclusions encompassed patients outside of the age range considered (under 13 and over 70 years).

Materials for the study were drawn from patients' surgical records and the associated histopathological findings post-resection. The methodology revolved around collating a comprehensive dataset that included both neoplastic and non-neoplastic lesions, as well as classifying them into benign or malignant categories.

Data were meticulously collected, encompassing demographics, clinical symptoms, radiological and cytological findings, surgical management, and postoperative outcomes. The analysis focused on constructing a comprehensive clinicopathological profile, reviewing imaging findings for nodule characteristics, and ensuring the accuracy of cytological diagnosis, which is done by assessing the concordance between the cytological findings and the final pathological diagnosis. All patients primarily underwent total thyroidectomy, and surgical details, along with the postoperative course, were closely documented. Histopathological examination by experienced pathologists identified benign and malignant cases based on specific criteria such as capsular and vascular invasions. Furthermore, the study tracked postoperative care, including adjuvant therapy administration and long-term follow-up to assess recurrence and overall survival, adhering to the rigorous ethical standards set forth by the Institutional Ethics Committee.

Data management

The collected data were systematically categorized according to different age groups and genders using a predefined proforma. The recorded details included the nature of the lesion, patient demographics, and clinical outcomes. This dataset was subsequently entered into Microsoft Excel (Microsoft Corporation, Redmond, WA), and statistical analysis was conducted using SPSS software (IBM Corp., Armonk, NY). The qualitative data, represented as percentages, were primarily examined using the chi-square test for any significant associations or trends.

During the period from January 2018 to March 2023, we conducted a retrospective review and got 32 patients diagnosed with Hurthle cell tumors. For all patients, we obtained detailed thyroid function profiles, including FT3, FT4, thyroid-stimulating hormone (TSH), and serum thyroglobulin. In parallel, ultrasonography data were accrued for all participants in the study. Each patient underwent FNAC for a thyroid nodule or neck node evaluation. A reported finding of a "follicular lesion" on FNAC was considered indicative of a potential malignancy, prompting surgical intervention. In instances where cervical lymph node involvement was detected either via preliminary imaging or during surgical examination, cervical lymph node dissection was performed.

The tumors were histopathologically classified into "carcinoma" if evidence of vascular or capsular invasion was present, or "adenoma" in the absence of such invasion. Tumor size information, critical for comprehensive assessment, was typically sourced from the surgical specimens, supplemented by imaging reports and clinical examination notes. The initial tumor extent was defined in concordance with the TNM (tumor, node, and metastasis) staging classification standards. All diagnosed invasive carcinomas were managed post-surgery with radioactive iodine therapy. Cases exhibiting metastatic disease underwent repeated radioactive iodine treatments. Following the primary treatment protocol, every patient embarked on levothyroxine TSH-suppressive therapy to aid in postoperative management and reduce the risk of recurrence.

Ultimately, this approach was chosen to ensure a thorough and ethically compliant investigation into the clinicopathological attributes of Hurthle cell tumors, seeking to enhance the overall understanding and clinical management of these neoplasms.

## Results

**Figure 1:**
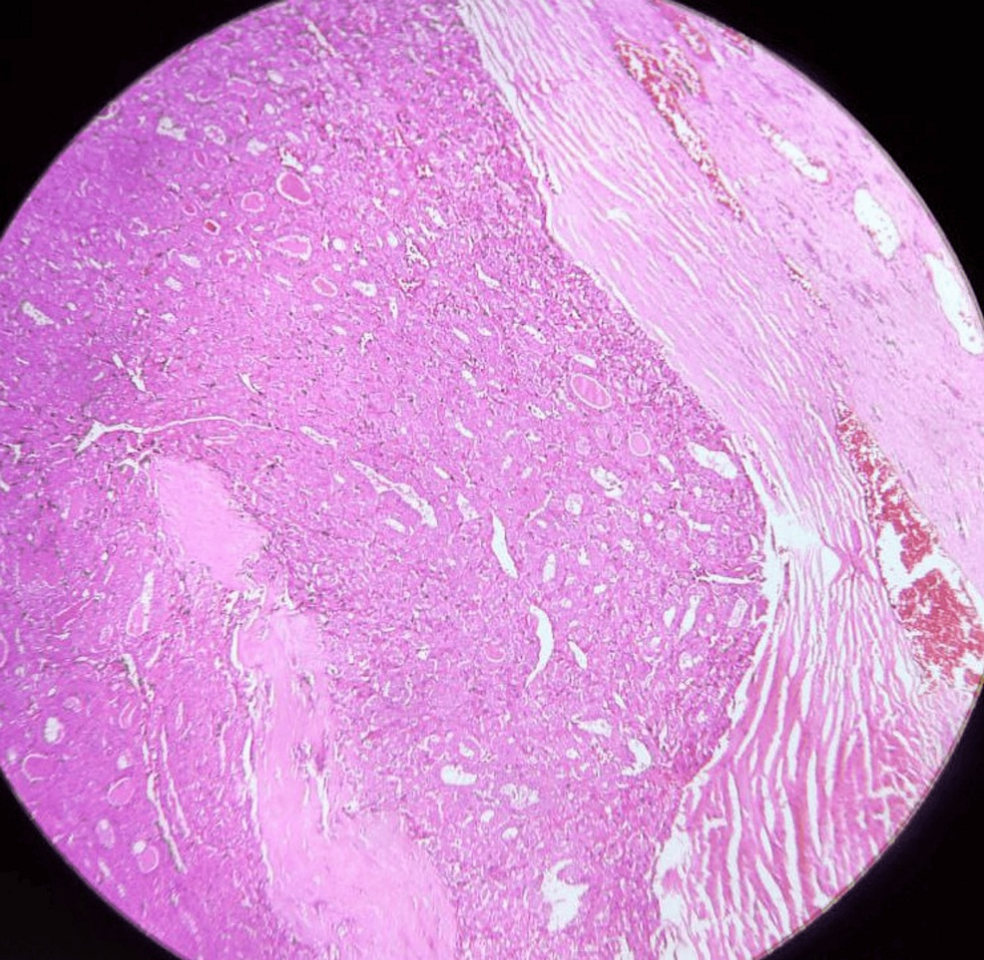
Low-power microscopy showing Hurthle cell with vascular and capsular invasion

The study included 32 patients, with 27 females and five males (Table 1). Out of these, 20 cases were diagnosed as Hurthle cell adenoma, and 12 cases were identified as Hurthle cell carcinoma upon final histopathological examination (Figure 1).

**Table 1:** Baseline characteristics of patients with Hurthle cell nodules * Thyroid Imaging Reporting and Data System (TI-RADS) category: 2 = not suspicious; 3 = mildly suspicious; 4 = moderately suspicious; 5 = highly suspicious.

Variables	Hurthle cell adenoma	Hurthle cell carcinoma
Number of patients	20	12
Male, n (%)	1 (20%)	4 (80%)
Female, n (%)	19 (70%)	8(30%)
Mean nodule size on histology (cm) (mean ± SD)	22.25 ± 1.09	30.2 ± 1.55
Bethesda cytologic diagnosis		
II	10	1
III	0	0
IV	10	11
VI	0	0
TI-RADS category*		
2	0	0
3	16	0
4	4	1
5	0	11

The median age of patients with adenoma was 43 years, ranging from 20 to 60 years, while the median age of patients with carcinoma was 52 years, ranging from 32 to 86 years. A total of 26 patients presented with neck swelling, one patient had a retrosternal extension, and in five patients, it was incidentally detected on neck imaging. No family history of malignancy was reported in any of the cases.

Prior to surgery, thyroid hormone levels were within the normal range for all patients. Out of the 32 patients studied, two exhibited elevated thyroglobulin levels preoperatively, with subsequent final reports confirming the diagnosis of Hurthle cell carcinoma. Conversely, the thyroglobulin levels in the remaining patients were within normal ranges. Ultrasonography (USG) revealed multiple nodules in both lobes in 15 cases, while 17 cases showed a solitary nodule. FNAC results indicated follicular neoplasm in 21 cases, colloid nodule in 10 cases, and one case exhibited lymphocytic thyroiditis.

**Figure 2:**
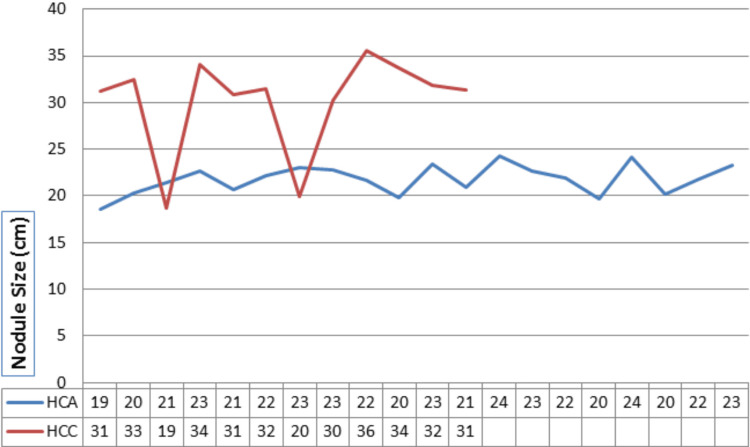
Nodule size in various subjects The Y-axis represents nodule size measured in millimeters, while the X-axis represents individual subjects. HCA: Hurthle cell adenoma; HCC: Hurthle cell carcinoma.

All 32 patients underwent total thyroidectomy. The mean diameter of the tumor was 23.3 mm in Hurthle cell adenoma patients and 30.2 mm in Hurthle cell carcinoma patients. In our study, thyroid nodules measuring less than 2 cm exhibited a 13% propensity for Hurthle cell carcinoma while for nodules more than 3 cm, it was 60% (p-value = 0.01; Figure 2).

Eight patients with Hurthle cell carcinoma had multifocal lesions. One patient had neck node involvement. Eight cases of Hurthle cell carcinoma showed capsular invasion and four cases showed vascular invasion.

**Figure 3:**
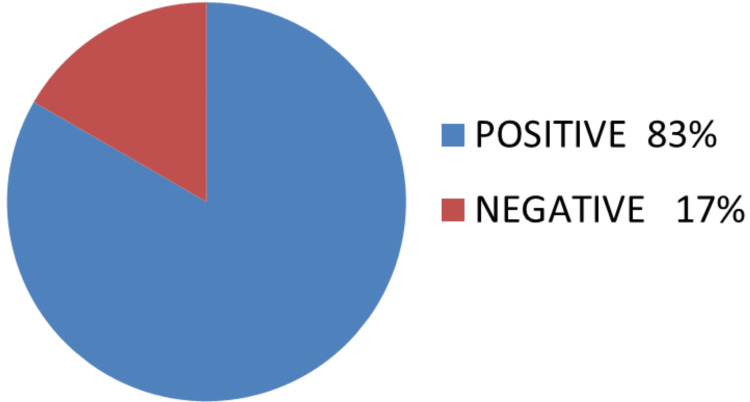
Hurthle cell carcinoma with I-131 uptake

None of the Hurthle cell invasive carcinoma cases showed distant metastases during the I-131 whole body examination. Ten cases showed radio uptake at the thyroid bed (Figure 3).

Throughout the follow-up period, all cases were meticulously monitored, and there was no evidence of metastasis or mortality related to Hurthle cell carcinoma and no cases of relapse were observed in any of the patients.

## Discussion

The demographic characteristics and clinical presentation of patients with Hurthle cell tumors provide valuable insights into the epidemiology and diagnostic challenges associated with this rare thyroid neoplasm. In our study, we observed a female predominance among the patients, with 84% females and 16% males, aligning with previous literature that suggests a higher prevalence in women [10,11]. The median age of patients diagnosed with Hurthle cell adenoma was 43 years, while those with carcinoma had a median age of 52 years. These findings are consistent with other studies that have reported the occurrence of Hurthle cell tumors in middle-aged to older adults [11]. With the Surveillance, Epidemiology, and End Results (SEER) dataset, there were several clinically significant predictors of poorer outcomes in Hurthle cell carcinoma, including male sex, increasing tumor size, and advancing patient age. Consequently, patients exhibiting these characteristics should be considered for more aggressive treatment strategies [12].

Previous reports on thyroid nodules with Hurthle cell cytology have highlighted nodule size as a crucial parameter influencing the probability of cancer in these nodules [13]. In our study, nodules smaller than 2 cm exhibited a cancer rate of 13%, with all cancers being minimally invasive. However, as nodule size increased, the likelihood of cancer also escalated. For nodules sized more than 3 cm, the cancer rate increased to 60%. The observed increase in cancer rate with larger nodule sizes underscores the importance of considering nodule size as a critical factor when assessing the preoperative risk of cancer in Hurthle cell nodules [14].

Differences in experiences have given rise to a controversial debate regarding the optimal treatment approach for Hurthle cell carcinoma. Some researchers advocate for conservative management of these cancers, while others advocate for more aggressive treatment strategies [15]. Total thyroidectomy was the most frequently performed surgical intervention in our study, consistent with previous literature advocating for aggressive resection for Hurthle cell carcinomas [16].

In the existing literature, there has been a widely accepted paradigm [17,18] stating that Hurthle cell thyroid carcinoma (HCTC) does not accumulate radioactive iodine (RAI). Consequently, reports describing effective radioiodine therapy for HCTC [19] were often regarded as anecdotal evidence [20]. However, contradicting this paradigm is the observation made by Samaan et al. [21] in patients with HCTC treated at MD Anderson Cancer Center and by a study conducted at the Institute of Oncology in Ljubljana, Slovenia, from 1972 to 2000 [12]. In our study, we found that 10 cases exhibited RAI uptake in the thyroid bed, corroborating previous findings in other studies that indicate Hurthle cells are capable of taking up iodine. These findings challenge the conventional understanding and warrant further investigation into the potential use of RAI therapy for certain subsets of HCTC patients.

Hurthle cell carcinoma is a relatively rare tumor, and consequently, there is limited large-scale data available concerning the long-term survival of patients with this condition. Existing research presents conflicting findings, with some studies suggesting a relatively benign course and prolonged survival, while others indicate aggressive behavior and poor expected survival rates [3].

Although the follow-up duration was relatively short in this study population, factors such as age, sex, size of the tumor, extra-glandular invasion, neck node involvement, and major capsular invasion were not found to have significant prognostic value in patients with Hurthle cell carcinoma. Interestingly, none of these risk factors were associated with case mortality or disease-specific mortality. The conflicting viewpoints highlight the need for further research and consensus to guide the best course of action for patients with Hurthle cell carcinoma.

Limitations of this study

Given the focused nature of the study, various limitations may be acknowledged. Firstly, the sample size of 32 cases limits the generalizability of the findings. The study is also confined to a single tertiary care center in South India, which may not reflect the broader population demographic and could influence the clinical presentation and management outcomes observed. Furthermore, the longitudinal follow-up was not done, potentially obscuring the long-term prognosis and recurrence rates. To strengthen future research, a larger, multicentric study with a prospective design and longer follow-up period is recommended to validate these findings and better understand the behavior of Hurthle cell tumors on a wider scale.

## Conclusions

Our study provides valuable insights into the clinicopathological profile of Hurthle cell tumors in South India. Despite their rarity, Hurthle cell tumors present diagnostic challenges and require multidisciplinary management. Our findings contribute to the existing literature and underscore the importance of accurate preoperative diagnosis, individualized surgical approaches, and informed decision-making regarding adjuvant therapies for better patient outcomes. Our study findings suggest that Hurthle cell carcinoma did not demonstrate aggressive behavior, which contrasts with observations reported by other researchers. The relatively indolent nature of Hurthle cell carcinoma observed in our study provides important insights into the disease's behavior in this particular cohort. Collaborative efforts among institutions and the accumulation of larger datasets will further enhance our understanding of these enigmatic tumors and aid in optimizing patient care.
